# Reelin signaling as a translational rheostat: linking synaptic homeostasis to neurodevelopment and neurodegeneration

**DOI:** 10.3389/fnmol.2025.1731914

**Published:** 2025-12-03

**Authors:** Murat S. Durakoglugil

**Affiliations:** 1 Department of Molecular Genetics, University of Texas Southwestern Medical Center, Dallas, TX, United States; 2 Center for Translational Neurodegeneration Research, University of Texas Southwestern Medical Center, Dallas, TX, United States

**Keywords:** Reelin, FMRP, APP, autism spectrum disorder, homeostatic plasticity

## 1 Reelin beyond development: the synaptic custodian

Reelin was first recognized as the secreted signal (D'Arcangelo et al., 1995) that orchestrates cortical lamination during embryonic development (Schiffmann et al., 1997). In the mature brain, Reelin remains highly expressed in GABAergic interneurons (Alcántara et al., 1998; Pesold et al., 1998) and continues to influence dendritic growth (Niu et al., 2004; Hamad et al., 2021b), synaptic organization, and neurotransmission. Upon binding to ApoER2 and/or VLDLR (Trommsdorff et al., 1999), it activates the adaptor Disabled-1 (Dab1) (Sheldon et al., 1997) via Src-family kinases (Arnaud et al., 2003; Bock and Herz, 2003), initiating PI3K/Akt/mTOR (Jossin and Goffinet, 2007) and MEK/ERK signaling pathways that govern cytoskeletal remodeling (Meseke et al., 2013; Dillon et al., 2017), receptor trafficking, and the maturation of NMDA receptor subunits (Qiu et al., 2006; Groc et al., 2007).

In addition to these canonical actions, Reelin directly modulates tau biology. Dab1 activation leads to inhibitory phosphorylation of GSK3β–a major tau kinase—thereby reducing tau hyperphosphorylation (Hiesberger et al., 1999; Beffert et al., 2002). Loss or reduction of Reelin results in disinhibited GSK3β activity and elevated tau phosphorylation, a mechanism confirmed in AD mouse models and human tissue (Rossi et al., 2020; Mouofo and Spires-Jones, 2023). This pathway provides a mechanistic link between Reelin deficiency and neurofibrillary tangle progression. Reelin's N-terminal and C-terminal domains also interact with non-canonical partners (Bock and May, 2016), including APP (Hoe et al., 2009), β1-integrins (Förster et al., 2002; Groc et al., 2007), and EphB receptors (Bouché et al., 2013). APP has emerged as an important convergence point between Reelin signaling and AD pathology: Reelin promotes APP trafficking and processing toward non-amyloidogenic pathways (Hoe et al., 2006), whereas Aβ oligomers disrupt Reelin receptor recycling and impair ApoER2-dependent phosphorylation of NMDA receptors (Durakoglugil et al., 2009). This establishes a bidirectional interaction: Reelin protects against Aβ toxicity, whereas accumulating Aβ progressively impairs Reelin signaling. ApoE genotype further modulates this system.

ApoE4, the strongest genetic risk factor for late-onset AD, interferes with ApoER2 recycling, reduces receptor surface expression, and diminishes Reelin's ability to phosphorylate Dab1 and stabilize glutamate receptors (Chen et al., 2010). Together, these mechanisms explain how ApoE4 carriers experience early synaptic vulnerability even in the absence of overt amyloid deposition. Overall, Reelin is increasingly recognized as a lifelong regulator of synaptic homeostasis whose dysfunction intersects directly with amyloid, tau, and ApoE biology. This revised perspective reframes Reelin not only as a developmental cue or plasticity modulator but as a central node in mechanisms of cognitive aging and neurodegenerative resilience.

## 2 Convergence with fragile X pathways

FMRP, the protein silenced in fragile X syndrome (FXS), binds specific mRNAs to suppress translation at synapses (Hale et al., 2021). Loss of FMRP leads to exaggerated group I metabotropic glutamate receptor (mGluR1/5) signaling (Bear et al., 2004), increased protein synthesis (Osterweil et al., 2010), and enhanced mGluR-dependent long-term depression (LTD) (Huber et al., 2002). In contrast, Reelin signaling facilitates NMDA receptor phosphorylation (Qiu et al., 2006) and opposes mGluR-LTD (Durakoglugil et al., 2021). These complementary actions position Reelin as a stabilizing influence against the excessive LTD observed in Fmr1-knockout models.

Importantly, Aβ oligomers converge on the same mGluR5-protein synthesis pathway implicated in FXS (Westmark and Malter, 2007). Aβ is known to enhance mGluR5-driven translation and elevate STEP61, a phosphatase that internalizes AMPA and NMDA receptors (Zhang et al., 2008). This mirrors—and in some cases exacerbates—the synaptic phenotype caused by loss of FMRP. Because Reelin reduces STEP61 levels (Durakoglugil et al., 2021) and blocks mGluR-LTD, its deficiency amplifies Aβ effects, while its presence mitigates them (Durakoglugil et al., 2009).

Tau pathology also intersects with these pathways. Hyperphosphorylated tau disrupts dendritic spine stability (Ittner et al., 2010; Avila et al., 2017), alters receptor trafficking, and impairs local translation, in part through disruption of dendritic transport and ribosomal integrity, mechanisms that parallel FMRP-dependent dysregulation. Reduced Reelin signaling, which normally restrains tau kinase activity (Hiesberger et al., 1999), further sensitizes synapses to mGluR- and Aβ-driven LTD. Thus, tau, Aβ, and FMRP deficits create a shared landscape of translational dysregulation that Reelin can partially buffer.

Together, these data reveal that Reelin and FMRP operate along a shared translational axis influenced by Aβ. Their convergence explains parallel phenotypes in neurodevelopmental and neurodegenerative disorders (Joly-Amado et al., 2023) and supports the need for an integrated biological model.

## 3 A translational rheostat integrating Reelin, FMRP, Aβ, tau, and ApoE

**Figure 1:**
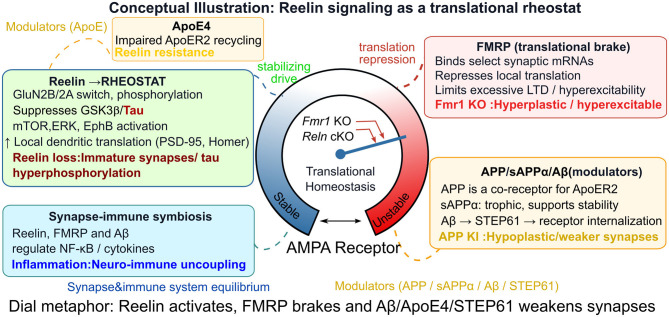
Conceptual model of Reelin signaling as a translational rheostat integrating synaptic, molecular, and neuroimmune cues. The central dial portrays the equilibrium of translational control as shaped by interacting stabilizing and destabilizing signals in neurons. Reelin signaling (Top left, green) via ApoER2/VLDLR receptors drives stabilization of the dendritic proteome, potentiation of NMDAR/AMPAR phosphorylation, and promotion of synaptic equilibrium. FMRP (top right, red) acts as a translational brake, repressing local protein synthesis and conferring resistance against excessive synaptic plasticity and hyperexcitability; its loss or knockout shifts the equilibrium toward instability. Modulatory molecules (bottom right, yellow) such as APP, sAPPα, and Aβ/STEP61 exert additional feedforward or repressive control. APP and its fragments link Reelin to neurodegeneration: sAPPα is trophic, while Aβ and its downstream effector STEP61 promote receptor internalization and weaken synapses. This entire system is further modulated by ApoE4, which induces Reelin resistance by impairing ApoER2 recycling, and Tau pathology, which is normally suppressed by the Reelin–GSK3β axis. Neuroimmune coupling (bottom left, blue) incorporates the influence of glial cytokines and NF-κB signaling on the synaptic rheostat, illustrating how chronic inflammation or immune modulation impacts synaptic stability. The central needled dial illustrates the opposing effects: stabilizing drive from Reelin versus repression/destabilization from FMRP, Aβ/STEP61, and ApoE4, with genetic perturbations directing the needle toward “stable” or “unstable” translational states. Color-coded annotation and dashed connectors identify each regulatory pathway and its direction of action.

The translational rheostat model proposes that synaptic stability arises from a balance of opposing forces: Reelin-driven enhancement of translation and receptor phosphorylation versus FMRP-mediated repression of protein synthesis (Figure 1). The original framework emphasized developmental and ASD-related mechanisms (Lammert and Howell, 2016); however, new evidence demonstrates that AD-related processes—Aβ, tau, and ApoE genotype—fit directly and mechanistically within this rheostat. Aβ integrates into the rheostat as a destabilizing input. By binding to mGluR5 and PrP^c^, Aβ oligomers amplify local translation, increase STEP61, and promote AMPA/NMDA receptor endocytosis. These actions push the rheostat toward an overly depressed, hypoplastic state. Reelin counters this by activating Src kinases (Bock and Herz, 2003), restoring receptor phosphorylation (Weeber et al., 2002) and suppressing STEP61 accumulation (Durakoglugil et al., 2021). Thus, Reelin acts as a synaptic antagonist to Aβ, shifting the rheostat away from pathological LTD.

Tau phosphorylation adds another destabilizing force. When Reelin signaling is reduced—through genetic deficiency, ApoE4, or chronic Aβ exposure—GSK3β inhibition weakens (Beffert et al., 2002), increasing tau phosphorylation. Hyperphosphorylated tau disrupts microtubule dynamics, spine stability, and receptor localization, further shifting the rheostat toward synaptic weakening. Reelin's normal role in restraining tau pathology provides a mechanistic bridge between FXS-like synaptic instability and AD-related tangle accumulation. ApoE genotype modulates the rheostat's sensitivity. ApoE4 impairs ApoER2 trafficking, reduces surface receptor levels (Chen et al., 2010), and dampens Reelin-mediated signaling. As a result, the stabilizing influence of Reelin is weakened, allowing Aβ and mGluR5 pathways to dominate. In contrast, ApoE2 supports more efficient receptor recycling, maintaining Reelin's stabilizing position on the rheostat. This may explain why the same molecular perturbations (e.g., Aβ exposure) produce greater functional consequences in ApoE4 carriers. Together, these components define a shared synaptic mechanism that spans neurodevelopmental disorders (FXS) and neurodegenerative diseases (AD). This expanded rheostat explains how Reelin supplementation can rescue FMRP-related phenotypes (Morrill et al., 2022) and why Reelin resilience variants protect against AD progression (Lopera et al., 2023).

## 4 The synaptic–immune interface

Reelin and FMRP modulate not only synaptic translation but also neuroimmune tone. Reelin can activate NF-κB signaling and regulate leukocyte adhesion, implicating it in vascular–immune interactions (Ding et al., 2016). FMRP, in turn, governs cytokine translation and inflammatory responses in microglia (Parrott et al., 2021). Other studies also link exaggerated microglial protein synthesis to autism-like phenotypes (Xu et al., 2020). In Alzheimer's disease (AD), Aβ oligomers strongly stimulate microglial activation, complement signaling, and cytokine release (Heneka et al., 2015), driving a feedback loop that disrupts synaptic pruning and spine maintenance (Rao et al., 2012).

In this context, recent research places Reelin in peripheral inflammation: Hepatocyte- derived Reelin increases leukocyte adhesion and NF-κB activation especially in vascular conditions like atherosclerosis, rheumatoid arthritis, and multiple sclerosis (Calvier et al., 2020, 2023, 2024; Alexander et al., 2023). FMRP normally restrains translation of inflammatory regulators; its loss exaggerates microglial reactivity and cytokine release (Hodges et al., 2020; Parrott et al., 2021; Dahl et al., 2022). There is evidence for association between maternal infections during pregnancy and increased risk of autism in the child later in life (Zerbo et al., 2015; Zawadzka et al., 2021). This inflammatory state, which contributes to synaptic loss in adults (Rao et al., 2012; Reive et al., 2024), may also dysregulate Reelin homeostasis in newborns.

## 5 Temporal and cellular context

Developmental and adult Reelin functions are mechanistically distinct. During embryogenesis, Reelin establishes laminar architecture; in adulthood, it maintains synaptic integrity. Reeler mice show severe neuronal migration defects and early lethality (Hamburgh, 1963), while heterozygous or conditional models exhibit subtle synaptic and behavioral phenotypes (Niu et al., 2004; Lane-Donovan et al., 2015). This temporal dichotomy indicates that Reelin serves as both architect and maintenance engineer.

Cell-type specificity adds another layer: interneuron-derived Reelin modulates oscillatory synchrony (Che et al., 2018; Iannone and De Marco García, 2021; Reive et al., 2024). Complementary work demonstrated that early postnatal Reelin signaling also governs cell-type–specific differentiation and network maturation, particularly via entorhinal stellate cells that drive perforant-path development (Hamad et al., 2024a,b). While early postnatal Reelin loss causes dendritic growth abnormalities, calcium dysregulation, and receptor imbalance (Hamad et al., 2021a,b), adult-onset deletion yields only minor changes (Lane-Donovan et al., 2015), emphasizing a restricted window of sensitivity. Finally, age-dependent declines in Reelin or ApoER2 have been linked to cognitive aging and Alzheimer's progression (Chin et al., 2007; Avila et al., 2017).

## 6 A systems view of synaptic homeostasis

From a systems perspective, the Reelin–FMRP–APP–Aβ-tau–ApoE axis forms an interconnected feedback network regulating dendritic translation and receptor trafficking (Figure 1).

- **Activation:** Reelin binds ApoER2/VLDLR (Trommsdorff et al., 1999), to stimulate Dab1 (Sheldon et al., 1997), Src (Bock and Herz, 2003), PI3K/Akt/mTOR (Jossin and Goffinet, 2007), and ERK pathways (Lee et al., 2014), enhancing local translation required for LTP (Beffert et al., 2005) and promoting receptor phosphorylation (Qiu et al., 2006).
- **Brake:** FMRP binds select dendritic mRNAs (Hale et al., 2021) to prevent excessive protein synthesis, guarding against synaptic overexcitation (Huber et al., 2002; Osterweil et al., 2010).
- **Degenerative Modulation:** Aβ oligomers increase STEP61, drive AMPA/NMDA receptor internalization, and skew the system toward LTD (Zhang et al., 2008). Reelin directly counteracts this Aβ-driven toxicity (Durakoglugil et al., 2009; Lane-Donovan et al., 2015).
- **Structural Destabilization:** Tau hyperphosphorylation disrupts cytoskeletal architecture (Dillon et al., 2017) and receptor trafficking, further weakening synaptic stability (Wang and Mandelkow, 2016).
- **Genetic Susceptibility:** ApoE4 impairs receptor recycling and reduces Reelin efficacy (Hiesberger et al., 1999) shifting the balance toward synaptic vulnerability (Chen et al., 2010).

Balanced Reelin and FMRP activity stabilize the network, whereas deficiencies in either—compounded by Aβ, tau, or ApoE4—produce hypoplastic (Liu et al., 2001) or hyperdepressed synaptic states characteristic of ASD, FXS, and AD (Bleuzé et al., 2021).

## 7 Translational and therapeutic perspectives

This integrated model highlights shared therapeutic targets across FXS and AD. Reelin supplementation has shown promise in rescuing synaptic and cognitive deficits in AD models (Pujadas et al., 2014). Recent findings also reported that a single intracerebroventricular injection of the central Reelin fragment (R3456; repeats 3–6) can ameliorate behavioral deficits in *Fmr1*-knockout mice (Morrill et al., 2022).

Thus, enhancing Reelin signaling—via recombinant protein, fragment supplementation, gene therapy, or small-molecule agonists—may restore synaptic stability in disorders marked by hypofunction. However, Reelin signaling extends beyond the canonical ApoER2/VLDLR–Dab1 cascade. Non-canonical pathways—including ERK activation and interactions with EphB receptors—are well characterized (Bouché et al., 2013; Lee et al., 2014), suggesting a broader receptor network operating at synapses. Notably, several transmembrane proteins, including APP, associate with Dab1 to integrate these signals (Trommsdorff et al., 1998; Hoe et al., 2006). Because the R3–6 fragment lacks the N- and C-terminal domains needed to engage such non-canonical partners, it likely restricts signaling to the canonical ApoER2/VLDLR route. Consequently, this fragment may compete with full-length Reelin for receptor engagement and thereby limit non-canonical signaling; interfering with the cooperative signaling required for optimal synaptic function. In neurodevelopmental conditions such as ASD, where alterations in Reelin processing or receptor engagement occur, the equilibrium between canonical and non-canonical signaling could therefore critically shape synaptic and behavioral outcomes.

Conversely, targeted inhibition of overactive pathways such as mGluR5, ERK, or tau kinases may counteract hyperplastic (FXS like) or destabilized (AD like) states. STEP61 inhibitors, already effective in AD models, represent a promising convergence point because STEP61 dysregulation appears in both Aβ-driven LTD and FMRP-deficient synapses (Goebel-Goody and Lombroso, 2012; Bagwe et al., 2023). Reelin's ability to suppress STEP61 (Durakoglugil et al., 2021) reinforces this therapeutic angle. Given that both Reelin and FMRP regulate microglial translation and immune tone, therapies combining synaptic and immune modulation may yield synergistic benefits.

Plasma and cerebrospinal Reelin concentrations are generally reported to be reduced in ASD and schizophrenia but variably altered in Alzheimer's disease (Impagnatiello et al., 1998; Joly-Amado et al., 2023). Interestingly, in a group of boys with ASD plasma Reelin levels were elevated more than thirty-fold attributed to increased proportions of Reelin dimers (Cuchillo-Ibanez et al., 2020). Such peripheral signatures may serve as biomarkers to stratify patients and monitor target engagement. Conceptually, reframing Reelin as a *translational rheostat* rather than a purely structural cue would unite the developmental, psychiatric, and degenerative mechanisms under a single regulatory principle.

## 8 Conclusion

Reelin's influence extends from neuronal migration to the coordinated regulation of translation, receptor composition, and immune balance across the lifespan. Acting through ApoER2/VLDLR and intersecting with FMRP, APP, Aβ, tau, and ApoE genotype, Reelin functions as a molecular rheostat maintaining synaptic homeostasis. Its deficiency yields a phenotype inverse to FMRP loss—reduced ERK/mTOR signaling, weakened synapses (Liu et al., 2001; Lee et al., 2014), elevated STEP61 (Durakoglugil et al., 2021), and enhanced vulnerability to Aβ (Lane-Donovan et al., 2015) and tau pathology (Lopera et al., 2023)—whereas FMRP loss causes dysregulation of mTOR signaling (Huber et al., 2015) and hypertranslation (Osterweil et al., 2010). ApoE4 exacerbates these vulnerabilities by impairing Reelin receptor recycling and weakening Dab1 signaling (Hiesberger et al., 1999). By situating amyloid, tau, and ApoE biology within the same translational framework that governs FXS, this expanded model provides a unified mechanistic lens through which neurodevelopmental (Folsom and Fatemi, 2013) and neurodegenerative disorders (Joly-Amado et al., 2023) can be understood. Restoring the delicate balance of various Reelin fragments in the brain may offer convergent therapeutic avenues to stabilize synaptic and immune function in conditions spanning ASD to Alzheimer's disease.
